# Emerging polymorphisms in falciparum Kelch 13 gene in Northeastern region of India

**DOI:** 10.1186/s12936-016-1636-4

**Published:** 2016-12-03

**Authors:** Neelima Mishra, Ram Suresh Bharti, Prashant Mallick, Om Prakash Singh, Bina Srivastava, Roma Rana, Sobhan Phookan, Hardev Prasad Gupta, Pascal Ringwald, Neena Valecha

**Affiliations:** 1 National Institute of Malaria Research, Sector 8, Dwarka, New Delhi, 110 077 India; 2 Global Malaria Programme, Geneva, Switzerland

**Keywords:** Malaria, Artemisinin resistance, K13 gene, PF3D7_1343700, *Plasmodium falciparum*

## Abstract

**Background:**

Recent reports of emergence and spread of artemisinin resistance in the Southeast Asia region, including Myanmar, pose a greater threat to malaria control and elimination in India. Whole genome sequencing studies have associated mutations in the K13 propeller gene (*k13*), PF3D7_1343700 with artemisinin resistance both in vitro and in vivo. The aim of the present study was to find the *k13* gene polymorphisms in *Plasmodium falciparum* parasites from the three sites in the Northeast region of India, bordering Bangladesh and Myanmar.

**Methods:**

A total of 254 samples collected during 2014–2015 from Tripura, Mizoram and Arunachal Pradesh states in the Northeast region of India were used to obtain the full-length *k13* gene sequences.

**Results:**

Three non-synonymous (NS) mutations: two in the propeller region, namely at codon 446 and 578, were observed besides one at codon 189 in the non-propeller region. The treatment outcome was not affected by these mutations at any of the sites. In addition, microsatellite variation in the N-terminus of the *k13* protein was observed at all the study sites.

**Conclusion:**

This is the first study to document the presence of F446I NS mutation in the *k13* propeller region from Changlang district, Arunachal Pradesh, a site adjoining the Indo-Myanmar border region, where this mutation is highly prevalent. In addition, NS mutation A578S has been observed only at Lunglei district, Mizoram, a site bordering Bangladesh and K189T mutation with relatively higher frequency in Mizoram and Tripura states. The presence of F446I mutation in a region close to the Myanmar border is notable. Considering the spread of anti-malarial drug resistance from Southeast Asia to the Northeast region of India in the past, there is an urgent need to undertake systematic mapping studies to ascertain the role and extent of this mutation in artemisinin resistance in this region of country.

## Background

Artemisinin resistance has been detected in Thai-Cambodia border, Vietnam and west Myanmar, in Southeast Asia (SEA). Delayed parasite clearance was first observed in falciparum malaria patients in Cambodia in 2008 [1]. Later, the reduced in vivo susceptibility to artesunate was reported from Cambodia in 2009 [2]. Considering the spread of anti-malarial resistance from SEA to the Northeast (NE) region of India, there is an urgent need to monitor the presence of artemisinin resistance in falciparum malaria in the border areas of the country.

In the NE region, artesunate plus sulfadoxine-pyrimethamine (AS + SP) was replaced by artemether-lumefantrine in 2013 due to high treatment failure with AS + SP in falciparum malaria during 2012 [3]. However, AS + SP remains the first-line therapy for uncomplicated falciparum malaria in the rest of the country [4].

Recently, mutations in the Kelch propeller protein, encoded by the PF3D7_1343700 gene, have been linked to artemisinin resistance, both through in vitro and in vivo studies [5]. Accordingly, the WHO definition of suspected artemisinin resistance includes cases with the presence of >5% of patients carrying *k13* resistance associated single nucleotide polymorphisms (SNPs) while for confirmed resistance, the *k13* mutants should be associated with either persistent parasitaemia on day 3 or longer parasite clearance time [6]. *k13* propeller SNPs have been investigated in different countries to detect the presence of resistant parasites. To date, resistance associated multiple mutations in the *k13* gene have been described in SEA particularly in the Greater Mekong region, mainly in propeller region and a few novel mutations [5–10].

Mapping of Kelch propeller region across Indian states over time has been done in 2014 where four non-synonymous (NS) mutations were detected in the propeller region. Out of these four NS mutations, three were from the NE region in the country, and one from Jalpaiguri, West Bengal, which connects the NE with the rest of India. The four NS mutations did not correlate with treatment outcome in the patients except one (G533A) from Gomati Tripura, where the outcome was late treatment failure on day 28 [11]. Presence of these four NS mutations prompted the focus in the NE region, and the *k13* gene was sequenced in 254 samples using Sanger sequencing method. All mutations observed in the study were validated with another PCR and by resequencing *k13*.

## Methods

### Collection of samples and DNA extraction

**Fig. 1:**
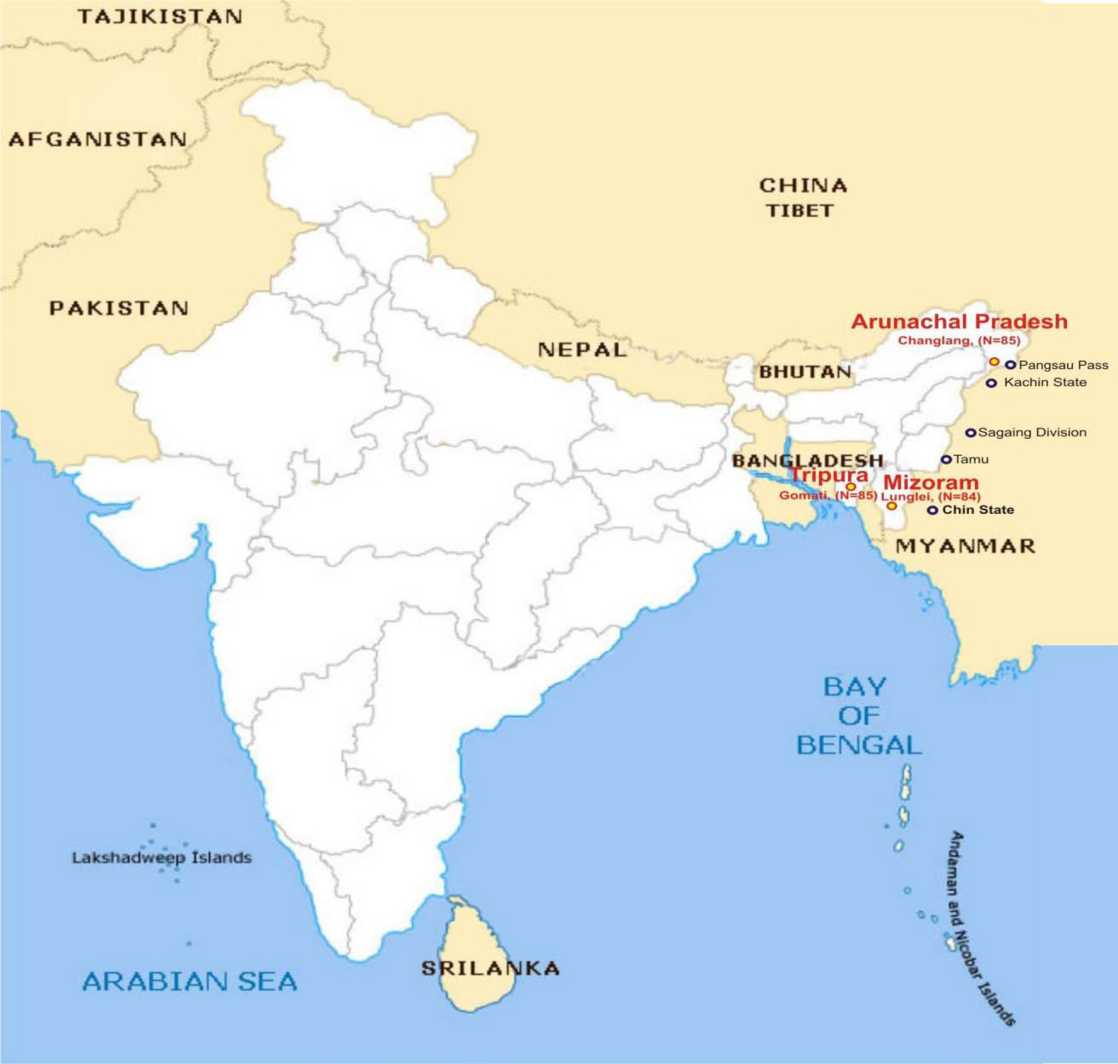
Details of study sites in Northeastern region in India

Patients were enrolled in therapeutic efficacy studies of artemether-lumefantrine in falciparum malaria at three study sites located in the NE region of India across international borders and were followed as per WHO guidelines [12]. Out of these study sites, two were located at the Indo-Bangladesh and one at Indo-Myanmar border (Fig. 1). Directly observed treatment of quality assured drugs supplied by the WHO was given. Finger-prick blood spots were obtained on Whatman filter paper strips (3 mm) from patients on day 0 and were analyzed for Kelch mapping.

### *k13* propeller gene amplification and sequencing analysis

Genomic DNA was extracted from dried blood filter using QIAamp^®^ DNA Mini Kit (QIAGEN, Germany), according to manufacturer’s instructions. The *k13* gene from all samples were amplified by PCR and the PCR products were sequenced (Macrogen, South Korea) as per protocols reported previously with modifications [5]. The initial primary PCR, amplifying the spanning region 53–2126 bases of the *k13* open-reading frame was carried out using high-fidelity Phusion PCR master mix (New England Biolabs, USA) with primers K13c53-F (5′-TGACGTATGATAGGGAATCTGG-3′) and K13c2126-R (5′-CCAAGCTGCCATTCATTTGT-3′). Three nested PCR reactions were performed (fragment 1, fragment 2 and fragment 3) amplifying fragments approximately 650–855 bases of the *k13* gene using primers K13c53-F(see above), K13c719-R(5′-TCTCGAATAAAATTCATTTGTGTCTT-3′), K13c614-F (5′-TTGAAACGGAATTAAGTGATGC-3′), K13c1464-R(5′-CAATACAGCACTTCCAAAATAAGC-3′), K13c1344-F(5′-AGGTGGATTTGATGGTGTAGAA-3′), and K13c2126-R (see above) respectively.

Briefly, 25 μl PCR reaction mix contains 12.5 μl Phusion PCR Master Mix (NEB, USA), 1 μl of specific primers (10 μM stock), and 2 μl of DNA template. Reaction conditions consisted of an initial denaturation at 98 °C for 30 s followed by 35 cycles of 98 °C for 10 s, 60 °C for 20 s and 72 °C for 30 s, and a final extension step for 10 min at 72 °C. Alignment of DNA sequences was performed using MEGA 6.0 with the *k13* sequence of the 3D7 clone (PF3D7_1343700) retrieved from Plasmo DB as the reference.

## Results

A total of 254 *Plasmodium falciparum* samples were collected during therapeutic efficacy studies of artemether-lumefantrine (AL) in the NE region. The samples included in the study were from Arunachal Pradesh (n = 85), Tripura (n = 85) and Mizoram (n = 84) states (Fig. 1). The *k13* gene was successfully PCR amplified from all the samples. DNA sequence analysis of *k13* from the 254 clinical isolates of *P. falciparum* showed three NS and one synonymous mutations (Table 1). In addition, variations in the number of microsatellite repeat (ATA) were observed in samples from all the study sites corresponding to amino acid positions 137-142 of *k13* in 3D7 parasite (Table 2).

**Table 1:** Point mutations in *Plasmodium falciparum* *k13* gene from Northeast India

Study site	Sample size (no.)	K13 mutation description		
		K189T	F446I	A578S
Changlang, Arunachal Pradesh	85		1	
Lunglei, Mizoram	84	5		1
Gomati, Tripura	85	3		
Total	254	8	1	1

An empty cell indicates no point mutation

**Table 2:** Site-wise details of microsatellite repeat (N) in *k13* gene

Sites	Repeats at microsatellite locus				
	6 N (wild type)	7 N	8 N	9 N	10 N
AP (85)	52 (61.18%)	–	33 (38.82%)	–	–
MZ (84)	63 (75%)	1 (1.19%)	19 (22.61%)	–	1 (1.19%)
SL (85)	53 (62.35%)	–	26 (30.58%)	6 (7.06%)	–

Low frequency of NS mutations in the propeller region at codon F446I and A578S was observed with predominant wild type genotype in the majority of the samples. Interestingly, the NS mutations, at amino acid position F446I, which corresponds to nucleotide position T1336A, was observed in only one sample from Changlang district, Arunachal Pradesh. However, the frequency of this mutation in these Indian sites was low (1.2%). The other mutation at amino acid A578S in the propeller region corresponding to nucleotide position G1732T, has also been observed in one sample from Lunglei district, Mizoram, a site adjacent to Bangladesh.

Besides these NS mutations, one NS mutation at proximal end (upstream region) at amino acid K189T corresponding to nucleotide position A566C has also been observed at two sites adjoining Bangladesh border. These include five samples from Mizoram (5.9%) and three (3.5%) from Tripura (Table 1). This mutation has been observed at a relatively higher frequency with complete absence in samples from Arunachal Pradesh. Out of 254 samples, only one synonymous mutation at nucleotide position T355C was observed in four samples from Tripura and one sample from Mizoram corresponding to amino acid position 119L. No synonymous mutations have been observed in samples from Arunachal Pradesh. Interestingly, the clinical outcome of all of these samples with mutations was adequate clinical and parasitological (ACPR) response after AL treatment and follow-up of 42 days. None of the patients showed delayed parasite clearance or day 3 positivity, an indicator of artemisinin resistance in the study areas.

## Discussion

For large-scale surveillance, *k13* propeller polymorphisms have been identified as a useful molecular marker, which will help in detecting emerging artemisinin resistance in the region. More than 100 NS mutations, have been reported worldwide. *In vivo* and in vitro data have confirmed that the *k13* propeller mutations 493H, 539T, 543T, and 580Y are associated with artemisinin resistance while many others are candidate markers for artemisinin partial resistance. These include 441L, 446I, 449A, 458Y, 553L, 561H, 568G, 574L, and 675 V. Distinct alleles originating from different independent events of emergence have been observed in Southeast Asia. The Y493H, R539T, I543T, and C580Y mutants are highly prevalent in Cambodia [5]. Recently, F446I has been reported during a therapeutic efficacy study conducted by the Department of Medical Research Upper Myanmar and WHO at the border between Myanmar and India (Lin, pers. comm.) and was further confirmed during a survey conducted in Myanmar [10, 13]. In addition, this mutant has been reported from the border between Myanmar and China and Myanmar and Thailand [14–16].

Therefore, compared to the Thai-Cambodia region, the scenario of *k13* mutation is quite different in the regions neighbouring northeastern India, which comprises Myanmar in the north and Bangladesh in the south. The *k13* propeller mutations C580Y, M476I, A481 V, N458Y, and R539T, which have been shown to be significantly associated with day 3 parasitaemia, are only reported from the eastern border of Myanmar [14]. The C580Y mutation is highly prevalent in Cambodia, Myanmar and eastern and western Thailand, while the F446I mutation, which was first reported in 2012, is predominant in China-Myanmar border regions as well as in Myanmar [8–10, 17].

This is the first report of the *k13* polymorphism in the propeller region at F446I from Changlang district, Arunachal Pradesh, in northeast India. The F446I mutation has been found to be associated with delayed parasite clearance (PCT) in the adjoining areas of Myanmar, as well as in China [18]. However, no correlation of this mutation with treatment outcome or delayed PCT was observed in the present study. Similarly, F446I has been observed to be associated with intermediate rate of parasite clearance in Upper Myanmar region [19]. Additionally, it is interesting to note that this NS mutation has been observed in only single sample from Changlang district, Arunachal Pradesh, a site which shares a border with Myanmar where this mutation has recently been reported at a higher frequency [10, 13]. The frequency of this mutation in the Indian site was comparatively low (1.2%). The absence of F446I mutation in these sites, which are across the Indo-Bangladesh border, is a finding consistent with the absence of *k13* mutant parasites in Bangladesh and most of west Myanmar, where a low prevalence of *k13* mutations has been observed [20].

Besides F446I mutation, A578S mutation has also been reported from Mizoram, a site in India adjacent to Bangladesh [11]. However, this mutation has been detected at a lower frequency and no correlation with treatment outcome has been observed. In Bangladesh, A578S mutation in the *k13* propeller region has been reported, however, no data on clinical outcome are available [21]. A recent study has revealed that A578S mutation is commonly observed in Africa and is not associated with artemisinin resistance [22]. In addition, one NS mutation at proximal end (upstream region) K189T has been observed from two study sites adjoining the Bangladesh border at comparatively higher frequency in Mizoram and Tripura. A recent study at the China-Myanmar border has also shown prevalence of K189T mutation at lower frequency (1.6%) compared to a higher prevalence which was observed at Dakar, Senegal [18, 20]. Although the K189T mutation was observed at a comparatively higher frequency in two sites, no correlation with clinical phenotype was observed in the samples.

Except F446I mutation, the other mutations K189T and A578S in the *k13* gene have not been found to be associated with delayed parasite clearance or confirmed artemisinin resistance. The presence in lower frequency as background mutations in propeller region or in the proximal end has been documented in the past in SEA and in Africa [9]. However, their association with artemisinin resistance is still unclear [11]. Although these mutations have been reported from northwestern areas of Myanmar and China bordering Indian states, their association with treatment failure in Indian samples has not been reported so far. With no change in the phenotypic characters, it is difficult to ascertain whether or not these mutations have any role in treatment failure.

As per the published literature, *k13* NS propeller region mutations are not always associated with artemisinin resistance. In fact, such mutants can represent ‘passer-by’ genotypes: that is, they do not lead to the selection of the mutant *k13* genotype [23]. Also, it could be suggested that these mutations in *k13* arise de novo rather than through clonal expansion.

Previously, in northwestern areas of Myanmar bordering India, the majority of mutations in the *k13* gene included C580Y, F446I and P574L [10]. Of these, the highest prevalence was observed for C580Y mutation, followed by F446I and P574L respectively. These highly prevalent mutations were present in more than 56% of the total number of drug-resistant samples. However, no such correlation has been observed in this study.

Miotto et al. [8] observed that parasites with the most common *k13* mutant alleles (C580Y, I543T, R539T, and Y493H) were found in countries in SEA, indicating that cross-border movement of *P. falciparum* may have already occurred. Since the study sites were located near international borders, larger population movement across these border areas is possible, which may assist in the spread to a larger extent.

Earlier, a few NS mutations at codon G533A, S549Y, R561H and A578S have been reported from India, all from the Northeast, including one from Jalpaiguri, West Bengal [11], which has international borders with Bhutan and Bangladesh in the North and South, respectively. Except A578S, no such mutations have been observed in this study.

Although AL remains efficacious in the study region and these mutations are in low frequency, artemisinin combination therapy may pose positive selection on the parasites. The rate of transmission and the diversity of vector species in the northeastern region may also increase selection pressure on parasite strains. There is an urgent need for the rational use of anti-malarials against *P. falciparum* strains and for priority on strengthening anti-malarial monitoring and surveillance. Detailed clinical and laboratory investigations to complement sentinel surveillance, including different tools to monitor resistance, are required in this region.

## Conclusion

Arunachal Pradesh in India shares a border with Myanmar and is prone to anti-malarial drug resistance. Emerging kelch polymorphisms, particularly F446I mutation in low frequency, could be an indication of emerging resistance towards artemisinin, however, more detailed investigations are needed to confirm this. The two NS mutations A578S and K189T observed at relatively higher frequency in Mizoram and Tripura could be background mutations. Thus, the presence of F446I warrants the urgent need to undertake systematic mapping studies to ascertain the role of this mutations in artemisinin resistance in this region.

## Abbreviations

NS: Non-synonymous
SEA: Southeast Asia
NE: Northeastern region
AS + SP: Artesunate plus sulfadoxine-pyrimethamine
SNPs: Single nucleotide polymorphisms
AL: Artemether-lumefantrine
AR: Arunachal Pradesh
MZ: Mizoram
ACPR: Adequate clinical and parasitological
PCT: Parasite clearance time
NIMR: National Institute of Malaria Research
WHO: World Health Organization
GMP: Global Malaria Programme
